# Total flavonoids of Potentilla discolor alleviate type 2 diabetes by regulating gut microbiota and endogenous metabolites

**DOI:** 10.3389/fmicb.2026.1875128

**Published:** 2026-08-13

**Authors:** Xiaoni Kong, Jie Song, Jiaming Han, Yuhao Zhao, YunSong Yang, Shaoping Wang, Xiaoping Yang

**Affiliations:** 1 The Medical School, Shandong College of Traditional Chinese Medicine, Yantai, China; 2 Foundation of Institute of Traditional Chinese Medicine Health Industry, China Academy of Chinese Medical Sciences, Beijing, China; 3 ShanDong Medical and Pharmaceutical University, Yantai, China; 4 Taizhou Institute for Drug Control, Taizhou, China; 5 The 970rd Hospital of the Chinese People's Liberation Army Joint Logistics Support Force, Yantai, China

**Keywords:** endogenous metabolites, gut microbiota, Potentilla discolor, total flavonoids, type 2 diabetes

## Abstract

**Background and aims:**

Type 2 diabetes mellitus (T2DM) is a chronic metabolic disease with an high incidence rate worldwide. It has recently shown a trend toward affecting younger populations. Total flavonoids from Potentilla discolor Bunge (PB) are associated with antipyretic and antidiarrheal properties. Several studies have also reported that PB exhibits anti-T2DM effects. However, the interactions between PB, T2DM, and gut microbiota, and the underlying mechanisms responsible for PB-mediated regulation of gut microbiota, have not been fully elucidated. This study performed *in vitro* fermentation, *in vivo* fecal microbiota transplantation (FMT), and 16S rRNA high-throughput sequencing to determine the mechanism by which PB ameliorates T2DM, with specific focus on its effects on gut microbiota.

**Methods:**

Ultra-high-performance liquid chromatography coupled with high-resolution mass spectrometry (UHPLC-HRMS) was used to analyze the flavonoid components in PB. The db/db mice were used as the T2DM model to assess the therapeutic effects of PB. The relationships between PB, T2DM, and gut microbiota were analyzed by 16S rRNA sequencing technology, *in vivo* FMT, and *in vitro* fermentation experiments. Fecal non-targeted metabolomics was also used to elucidate the potential mechanisms.

**Results:**

In PB, we identified 41 compounds, including 8 flavonoids, 9 flavonols, and 2 dihydroflavonoids. PB effectively reduced the blood glucose and glycated hemoglobin (GHb) levels (*p* < 0.05), increased the levels of insulin and glucagon-like peptide-1 (GLP-1) (*p* < 0.05), and significantly alleviated T2DM-related damage in the liver, kidney, and pancreas in T2DM model mice (*p* < 0.05). The 16S rRNA sequencing results showed that Firmicutes and Bacteroidetes were the main phyla and Ligilactobacillus, Limosilactobacillus and Prevotellaceae-UCG were the key bacterial genera in the T2DM mice. Fecal non-targeted metabolomics results identified 15 differential metabolites between control, T2DM, and PB-treated T2DM mice. Metabolites such as isoindoline, ethyl 2-aminobenzoate, L-tyrosine, argininic acid, glutamine, dodecylbenzene, 1-octene, 2,3-pentanedione, benzohydrazide, maslinic acid 3-O-*β*-D-glucoside and 1-pentanol were significantly upregulated in the T2DM model group, whereas high-dose PB treatment (HPB) effectively reversed these effects and restored their composition to a normal range.

**Conclusion:**

The present study demonstrates that PB contains several flavonoid components, which ameliorate glucose metabolism and alleviate organ injury in T2DM mice by restoring gut microbiota homeostasis. Furthermore, identification of 15 differential metabolites by fecal metabolomics provides important information for further in-depth mechanistic research.

## 1 Introduction

Type 2 diabetes mellitus (T2DM) is a highly prevalent chronic metabolic disorder worldwide. It is characterized by insufficient insulin secretion and/or insulin resistance, resulting in persistent hyperglycemia (Hemmingsen et al., 2017; Richter et al., 2018). The number of individuals with T2DM is projected to rise from 345 million in 2019 to 540 million in 2045 (Schmidt et al., 2014). At present, there are several drugs for the clinical management of T2DM (Harreiter and Roden, 2023; Ojo, 2021), including statins and fibrates, which significantly improve T2DM but are associated with some adverse reactions (Dutta et al., 2023). Previous studies have shown that a variety of bioactive components including flavonoids, polysaccharides and polyphenols from natural products ameliorate aberrant glucose and lipid metabolism, alleviate insulin resistance, protect pancreatic *β*-cells, and suppress oxidative stress and inflammation (Ni et al., 2024; Liu et al., 2022; Liu et al., 2023).

Total flavonoids extracted from the dried whole herb of Potentilla discolor Bge. (Rosaceae) or PB are used in classic traditional herbal medicine for clearing heat and detoxification, hemostasis, antidiarrhea, and detumescence (Ji et al., 2022; Yang et al., 2023). PB alleviates diabetic nephropathy in mice by regulating the expression of GAS5 and miR-21; it also improves high-sugar diet-induced T2DM in *Drosophila melanogaster* via the JAK/STAT signaling pathway (Li et al., 2023). Flavonoids are recognized as the primary bioactive constituents of PB, but specific bioactive components remain poorly elucidated.

Glucagon-like peptide-1 (GLP-1) is an incretin hormone primarily secreted by intestinal L cells. It maintains glucose homeostasis by stimulating insulin secretion and suppressing glucagon release. Gut microorganisms modulate glucose homeostasis by regulating GLP-1 levels (Liu, 2024; Gomes et al., 2018). The gut microbiota is a vital microbial ecosystem in humans and is regarded as the human “second genome.” It plays an indispensable role in systemic energy metabolism, anti-inflammatory responses and intestinal barrier integrity (Smith et al., 2019; Liu and Yang, 2023).

Currently, 16S rRNA high-throughput sequencing is a widely used for the qualitative and quantitative analysis of gut microbial composition and their relative abundance and diversity. Several studies have reported varying degrees of gut dysbiosis in the T2DM rodent models. Moreover, remodeling of gut microbial homeostasis contributes to the alleviation of T2DM (Johnson et al., 2019; Li M. N. et al., 2024). Few studies have reported alterations in the gut microbial structure after treatment with PB water extract. In this study, we integrated *in vitro* fermentation, *in vivo* fecal microbiota transplantation (FMT), and 16S rRNA high-throughput sequencing to verify whether PB ameliorates T2DM by targeted modulation of gut microbiota composition and restoration of gut dysbiosis.

Fecal metabolomics, an important branch of multiomics analysis technology, can accurately identify changes in the types and concentrations of metabolites in feces. It has become a core tool for mining disease biomarkers and clarifying the mechanisms of disease onset and drug intervention (Deda et al., 2025; Song et al., 2024). Previous studies have reported that PB treatment regulates the composition and abundance of the intestinal microbiota related to T2DM. This suggests that its anti-T2DM effects may be achieved by regulating metabolism of the intestinal microbiota (Han et al., 2019). Therefore, this study conducted in-depth investigation of the metabolic products from the intestinal microbiota in T2DM.

## 2 Materials and methods

### 2.1 Materials

Metformin hydrochloride (MET-HCl) was purchased from Merck Pharmaceutical Manufacturing (Jiangsu) Co., Ltd. The kits for GLP-1, GHb, INS, hematoxylin–eosin staining, etc. were purchased from Beijing Solarbao Biotechnology Co., LTD. Gifu anerobic culture medium (GAM), anerobic gas bags, anerobic culture bags, vitamin K1, and chloroheme were purchased from Shandong Hehe Biotechnology Co., Ltd. in China. Mass spectrometry-grade methanol, formic acid, acetonitrile and ethanol were purchased from Kemiou Company in Tianjin, China.

### 2.2 Preparation of PB

PB were prepared in-house by the research team of the Shandong University of Traditional Chinese Medicine. A total of 500 g of Potentilla discolor was cut into pieces and extracted by heating reflux with 70% ethanol for 1.5 h twice. The first extraction used 10 volumes of 70% ethanol and the second extraction used 6 volumes of 70% ethanol. The extracts were filtered and combined. Ethanol was removed under reduced pressure until no alcohol smell remained. The solution was concentrated to obtain a crude extract. D-101 macroporous resin was used for purification. The column was packed with resin by wetting with ethanol. Then, it was rinsed with 95% ethanol and fully washed with ultrapure water to remove residual ethanol. After sample loading and adsorption saturation, the column was eluted with 3 column volumes (BV) of ultrapure water, followed by 3 BV of 70% ethanol at a flow rate of 0.5 mL cm-1. The 70% ethanol eluate was collected, concentrated under reduced pressure to remove ethanol, and vacuum-dried at 60 °C to obtain 9.94 g of brown powder. The total flavonoid content was determined using the sodium nitrite–aluminum nitrate–sodium hydroxide method. The flavonoid content was estimated to be 72.3%.

### 2.3 Qualitative analysis of PB by UHPLC–HRMS

#### 2.3.1 Preparation of test samples

A total of 1 g PB was dissolved in 10 mL methanol, followed by 30 min ultrasonication until full dissolution. Then 1 mL PB solution was retained for further detection.

#### 2.3.2 UHPLC–HRMS

The components of PB were analyzed by ultra-high performance liquid chromatography–high resolution mass spectrometry (UHPLC–HRMS). Briefly, the separation was performed in the Waters ACQUITY UPLC–HILIC column set at a temperature of 40 °C. The flow rate was 0.3 mL/min. The injection volume was 2.0 μL. The mobile phases were 0.1% formic acid aqueous solution and acetonitrile. The following gradient was used for mobile phase B: 0 ~ 3 min, 95%; 3 ~ 6 min, 90%; 6 ~ 18 min, 79%; 18 ~ 30 min, 74%; 30 ~ 40 min, 49%; 40 ~ 45 min, 35%; 45 ~ 50 min, 20%; 50 ~ 55 min, 95%.

The following protocol was used for HRMS condition: both positive and negative ionic modes were used; capillary voltage, 35 V; capillary temperature, 320 °C; auxiliary gas flow rate, 10 arb; auxiliary gas temperature, 350 °C; spray voltage, 3,500 V (+), 3,000 V (−); tube lens voltage, 110 V; sheath gas flow rate, 30 arb; collision energy, 30 eV. The mass spectrum scanning range was set as 70 ~ 1,050 m/z.

### 2.4 Treatment of db/db mice with PB

Thirty-two 6-week-old male db/db mice and eight 6-week-old C57BL/6 J mice of SPF grade and weighing 35 ± 5 g were purchased from Jinan Pengyue Laboratory Animal Breeding Co., LTD. (Laboratory animal number: SCXK (Lu) 20,190,003). They were housed in a SPF-level animal room maintained with a temperature of 24 ± 2 °C, relative humidity of 60 ± 5%, and 12/12-h light/dark cycle. This study was approved by the Animal Ethics Committee of Shandong Medical and Pharmaceutical University (Ethical Approval No.: 2023-004). After one week of adaptive feeding, 8 C57BL/6 mice were set as the control group (Con), and 32 db/db mice were randomly divided into the model group (Mod), metformin hydrochloride group (MET-HCl), low-dose PB group (LPB), and high-dose PB group (HPB) (*n* = 8 mice per group). Mice in the Con group and the Mod group received 0.1 mL of normal saline by gavage daily. The HPB group received 400 mg·kg^−1^ PB per mouse. The LPB group received 100 mg·kg^−1^ PB per mouse. The MET-HCl group received 200 mg·kg-1 metformin hydrochloride per mice. All mice were administered 0.1 mL of their corresponding doses by gavage once daily for four weeks.

### 2.5 Fecal microbiota transplantation

Thirty-two mice were included in the FMT experiment. Eight db/db mice were included as the model group (m) and twenty-four C57BL/6 mice were randomly divided into control (B), FMT, and FMTH groups (*n* = 8 mice per group). After successful modeling (m group), fresh fecal samples were collected, homogenized with sterile normal saline, and filtered through sterile gauze. The filtrate was subsequently centrifuged at 3500 rpm for 10 min. After discarding the supernatant, the pellet was resuspended in sterile normal saline. Then, FMT and FMTH group mice were administered the fecal solution (0.1 mL per day). The blood glucose differences between the B and FMT groups were estimated in real time during the transplantation process as a criterion for successful transplantation. Based on the results of preliminary dose screening (100 mg·kg^−1^ and 400 mg·kg^−1^), 200 mg·kg^−1^ PB was selected as the optimal dose for the FMT experiment.

### 2.6 Collection and preservation of biological samples

At the end of the experiment, all mice were fasted without food for 12 h, and then euthanized by cervical dislocation. Blood was collected from all groups of mice, placed in an EP tube coated with heparin sodium, and centrifuged at 3500 rpm for 10 min at 4.0 °C. The serum (supernatant) was harvested for analysis. The liver, kidney and pancreas of all mice were rapidly harvested after euthanasia and fixed in 4% paraformaldehyde solution.

Serum samples were analyzed to estimate the levels of GLP-1, glycated hemoglobin (GHb), and insulin using specific ELISA kits. Hematoxylin and eosin (H&E) staining was performed on the liver, kidney and pancreatic tissue sections. Immunofluorescence staining of pancreatic tissue sections was performed for glucagon and insulin. The samples from mice in sections 2.4 and 2.5 underwent the same experimental determinations. Liver tissue sections of mice listed in section 2.4 were also stained with Oil Red O.

### 2.7 The fecal fermentation of PB

Fresh feces was collected from the Con and Mod mice listed in section 2.4. Then, 1 g of feces from each group was mixed with 5 mL of sterile normal saline, homogenized thoroughly with a sterile glass rod, and filtered through sterile gauze to prepare fecal suspensions C (control) and M (model), respectively. Furthermore, 1 g of GAM broth powder was dissolved in 20 mL of deionized water and sterilized at 121 °C under 0.15 MPa for 15 min. After sterilization, vitamin K1 and hemin were added to the medium at a final concentration of 1.0%. The fecal suspensions were mixed with GAM medium at a ratio of 1:1. Eight parallel samples were prepared for each group. Four samples from each group were incubated with 6 mg PB and designated as CY and MY groups, respectively. All samples were placed in anaerobic bags to simulate an anaerobic environment and incubated in a shaker at 37 °C for 24 h.

### 2.8 16S rRNA sequencing of gut microbiome

Fecal samples from all experimental groups were collected, and bacterial genomic DNA was extracted using the CTAB method. Extraction blanks (lysis buffer without fecal material) and no-template PCR negative controls were set throughout DNA extraction and amplification to eliminate exogenous microbial contamination. The V3–V4 hypervariable region of the 16S rRNA gene was amplified with primer pairs 338F and 806R under standard PCR conditions. Qualified amplicons were used to construct sequencing libraries, which were quantified by Qubit 3.0 and sequenced on the NovaSeq 6000 PE250 platform.

Raw sequencing reads were quality-filtered with Trimmomatic, chimeric sequences were removed by USEARCH 10.0, and mouse host DNA fragments were filtered out by genome alignment. All samples were normalized to a unified rarefaction depth of 42,000 valid reads, and rarefaction curves were plotted to confirm adequate sequencing coverage. Clean reads were clustered into OTUs at 97% similarity, and taxonomic annotation was performed against the SILVA v138 database. Alpha diversity indices including Chao1 and observed OTUs were calculated in QIIME2 2020.6. Beta diversity based on Bray–Curtis distance was visualized via PCoA and NMDS, and PERMANOVA with 999 permutations was applied to test intergroup community differences.

Kruskal–Wallis H test combined with Benjamini–Hochberg FDR correction was used to screen differential taxa (FDR-*p* < 0.05). LEfSe analysis with an LDA threshold of 2.0 was conducted to identify group-specific biomarker bacteria. PICRUSt2 v2.4.0 was adopted to predict microbial KEGG functional pathways, and pathways with FDR-*p* < 0.05 and fold change > 1.2 were defined as significantly altered functional modules.

### 2.9 Fecal metabolomics

Briefly, 0.1 g freeze-dried fecal sample was extracted with methanol via ultrasonication, followed by centrifugation to collect supernatants. After nitrogen blow-drying, residues were reconstituted with methanol. A pooled QC sample mixed from all extracts was injected once per 10 test samples to monitor instrument stability, with acceptable criteria defined as retention time RSD < 0.2 min and peak area RSD < 15%. Solvent blank, medium blank and consumable blank samples were tested under identical LC–MS conditions to exclude exogenous contaminant signals.

All metabolic peaks were normalized by total ion current (TIC). Missing values were filled and extreme outliers were removed before multivariate analysis. Metabolite annotation strictly followed MSI four-level criteria: all 15 differential metabolites were Level 2 annotated via HMDB v5.0 and METLIN databases with mass tolerance ≤ 5 ppm by matching exact mass and MS/MS fragments.

SIMCA 14.1 was used to construct OPLS-DA models, and 20 permutation tests were performed to avoid overfitting. Metabolites with VIP > 1.0 and Benjamini–Hochberg FDR-adjusted *p* < 0.05 were identified as significantly differential metabolites. KEGG pathway enrichment analysis was carried out on MetaboAnalyst 6.0, and pathways with FDR-*p* < 0.05 were regarded as significantly enriched metabolic pathways.

### 2.10 Statistical analysis

Mass spectrometry data acquisition was performed using the Thermo Xcalibur software (version 2.2). Statistical data analysis was conducted using the GraphPad Prism 8.0 software. Student’s t-test was used for multiple comparisons between different groups. *p* value < 0.05 was considered statistically significant.

## 3 Results

### 3.1 Qualitative analysis of PB by UHPLC–HRMS

**Figure 1:**
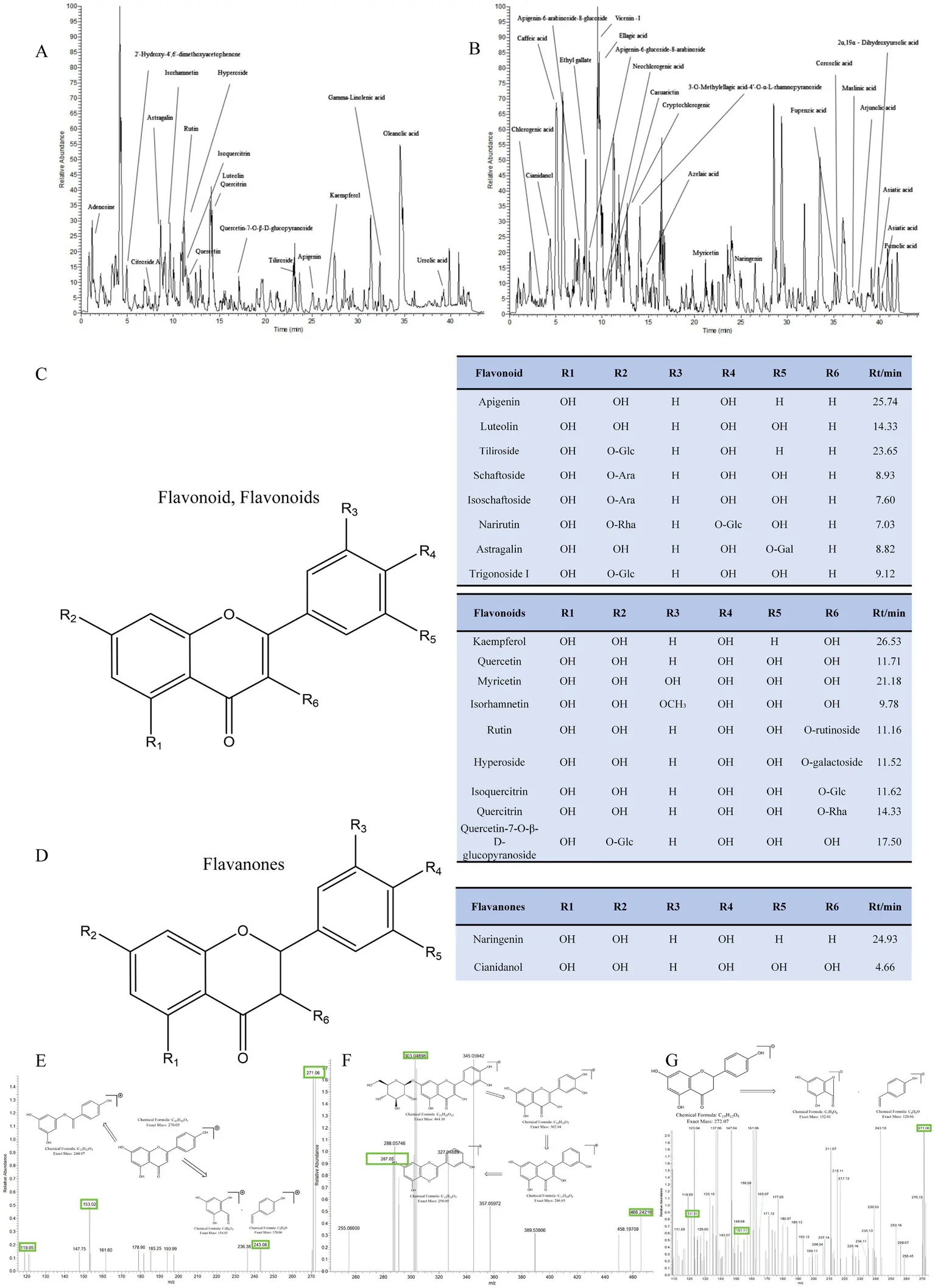
Chemical structures and retention times of compounds in PB: **(A)** Positive ion mode; **(B)** Negative ion mode; **(C)** Flavones and flavonol constituents; **(D)** Flavanone constituents; **(E)** Apigenin; **(F)** Quercetin-7-O-*β*-D-glucopyranoside; **(G)** Naringenin.

The full scans of PB in positive and negative ion modes were obtained using UPLC–HRMS (Figures 1A,B). Based on the analysis, 41 components were identified, including 8 flavonoids, 9 flavonols, and 2 dihydroflavonoids (Figures 1C,D). The other components are listed in Supplementary Table 1.

The flavonoid profile of PB included apigenin, luteolin, tiliroside, schaftoside, isoschaftoside, narirutin, astragalin, and trigonoside I. In the Electrospray Ionization (ESI)^+^ mode, the precursor ion of the compound with *m/z* value of 271.05942 was apigenin. Its molecular formula was C_15_H_10_O_5_ with a mass error of −2.508 ppm and elution time of 25.74 min. In the MS/MS spectrum, the ion at *m/z* 271 could be broken down into fragment ions at *m/z* 153 and *m/z* 119 due to the Retro-Diels-Alder (RDA) reaction. Therefore, the ion at *m/z* 271 could also lose -CO (28 Da) to produce a fragment ion at *m/z* 243 (Figure 1E).

The flavonols in PB included kaempferol, quercetin, myricetin, isorhamnetin, rutin, hyperoside, isoquercitrin, quercitrin, and quercetin-7-O-*β*-D-glucopyranoside. In the ESI^+^ mode, quercetin-7-O-β-D-glucopyranoside generated an ion at *m/z* 465.10275. The predicted formula was C_21_H_20_O_12_ with a mass error of −1.768 ppm and retention time of 17.5 min. In the MS/MS spectrum, the excimer ion at *m/z* 465 lost glucopyranoside (162 Da) to generate a fragment ion at *m/z* 303. Then, removal of one a carboxyl group (45 Da) generated a fragment ion with *m/z* 287; further loss of a carbon monoxide (CO) generated a fragment ion with an *m/z* 259 (Figure 1F).

PB contained dihydroflavonoids such as naringenin and cianidanol. Pyrolysis of naringenin is depicted as an example to illustrate the process of component identification. In the ESI^−^ mode, naringenin generated a precursor ion at *m/z* 271.06119 in the mass spectrum. The predicted molecular formula was C_15_H_12_O_5_ with a mass error of 0.307 ppm and elution at 24.93 min. In the MS/MS spectrum, the excimer ion at *m/z* 271 underwent RDA reaction to fragment ions at *m/z* 151 and *m/z* 119 (Figure 1G).

### 3.2 Pharmacodynamic evaluation of PB against T2DM

**Figure 2:**
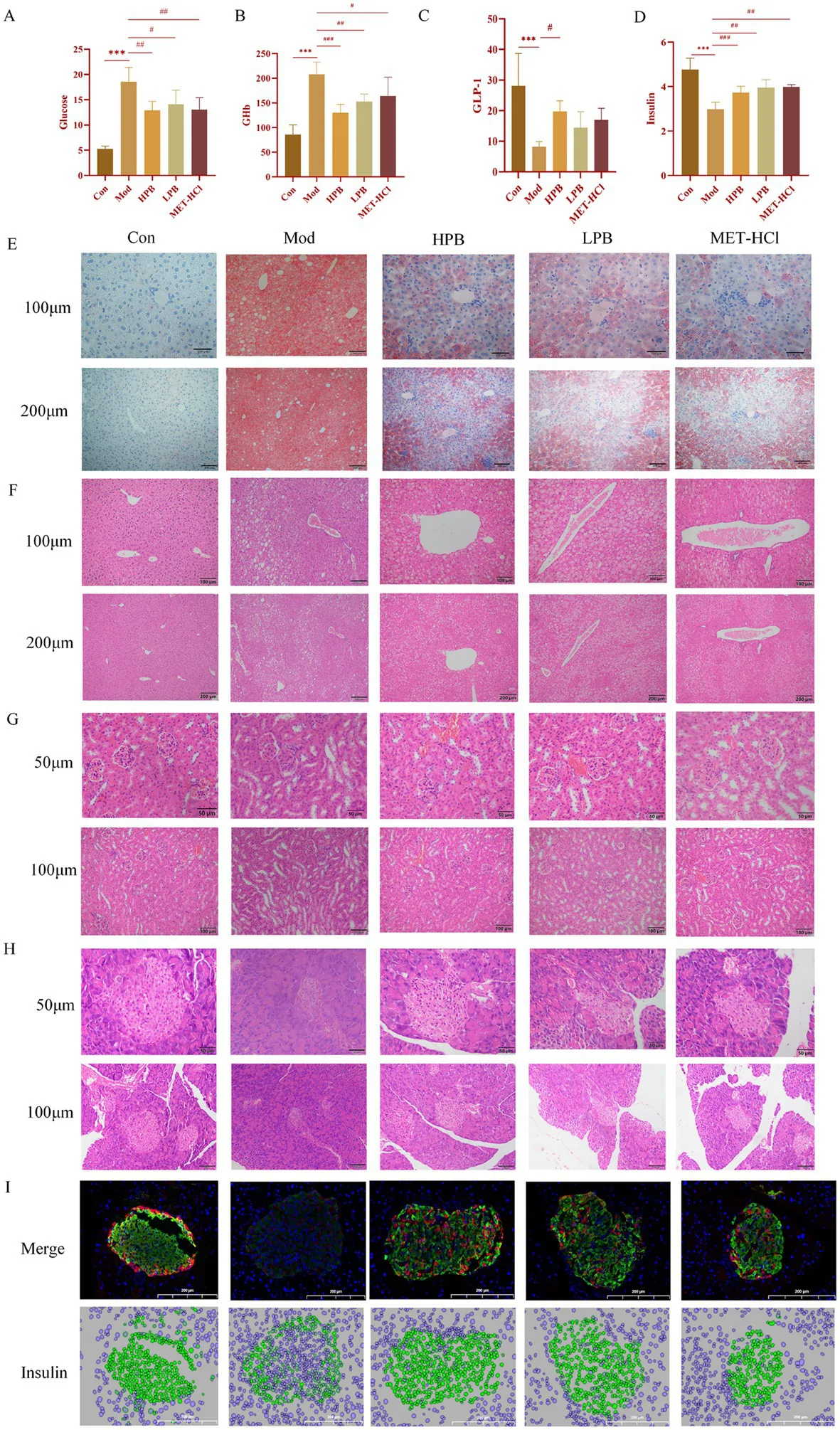
The result of PB alleviating T2DM. **(A)** Glucose. **(B)** GHb. **(C)** GLP-1. **(D)** Insulin. **(E)** Results of Oil Red O staining in liver. **(F–H)** HE staining results of liver, kidney, and pancreas. **(I)** Immunofluorescence results of pancreas. Immunofluorescence double staining of pancreas. Red: Insulin; Green: Glucagon. ***p < 0.001 vs. Con group; #*p* < 0.05, ##*p* < 0.01, ###*p* < 0.001 vs. Mod group.

During the experiment, all animals were allowed food and water without any restrictions. As shown in Figure 2, the blood glucose levels of Mod mice increased gradually and were significantly higher than those of the Con mice (*p* < 0.05). In contrast, blood glucose levels of the HPB, LPB, and MET-HCl group mice were significantly lower than those of the Mod group mice (*p* < 0.05, Figure 2A). Compared with the Con group, the Mod group mice showed significantly higher levels of GHb as well as lower GLP-1 and insulin level (*p* < 0.05, Figures 2B–D). This demonstrated successful establishment of the T2DM model with insulin resistance. However, PB administration effectively reduced the level of GHb and contributed to the improvement of persistent hyperglycemia. Furthermore, PB up-regulated GLP-1 and insulin and reversed pathological hyperinsulinemia, thereby alleviating insulin resistance in the diabetic mice.

Histological examination of liver sections from the T2DM mice was conducted using Oil Red O and H&E staining. The findings demonstrated disordered arrangement of hepatocytes with indistinct cell contours in the Mod group. A substantial number of fat vacuoles and lipid droplets were present in the cytoplasm. Furthermore, hepatocyte degeneration and necrosis was also observed. Many red lipid droplets were diffusely distributed and aggregated in the liver tissue of the Mod group. This indicated a significant degree of lipid deposition. However, these pathological effects were significantly mitigated in mice treated with PB (Figures 2E,F).

In the H&E-stained sections of renal tissue, the Mod group demonstrated obvious renal injury, including glomerular mesangial hyperplasia, swelling and degeneration of renal tubular epithelial cells, accompanied by significant renal interstitial congestion, edema, and inflammatory cell infiltration. These pathological changes were significantly alleviated in the HPB and MET–HCl groups, with a tissue structure comparable to the Con group; the LPB group only showed partial improvement (Figure 2G).

The pancreatic tissue sections were subjected with H&E and immunofluorescence staining. In the Mod group, the volume of islets were significantly reduced with obvious atrophy. The boundary between islet tissue and acinar cells was blurred, the cell arrangement was disordered, the number of islets were reduced, and some cells underwent vacuolar degeneration. The expression of target proteins was significantly decreased and the fluorescence signals were sparse and disordered. In the HPB, LPB, and MET–HCl groups, the pancreatic structure appeared normal; the capsule remained intact; and the distribution and morphological structure of the islets was comparable with the Con group (Figure 2H).

Double immunofluorescence staining with glucagon and insulin was performed to assess pancreatic *β*-cell positive ratio. The pancreatic β-cell positive ratio was high in the Con group (65.61%), but significantly reduced to in the Mod group (20.35%), thereby indicating severe islet β-cell injury and dysfunction. The β-cell positive ratio in the HPB group was 63.46%, nearly reaching the normal level; LPB group also demonstrated good protective effect of PB. In contrast, MET-HCl showed a weaker therapeutic effect. These findings suggest that PB promotes pancreatic β-cell proliferation and differentiation, repairs islet damage, and restores islet functional homeostasis in the model mice (Table 1; Figure 2I).

**Table 1:** Quantitative analysis of pancreatic immunofluorescence.

Name/PN	DAPI TCS	Tissue area	Number of positive cells	Positive cell density	Positive cell ratio	Change trend
Con IF Glu + Insulin	628	0.1668	412	2,470	65.61%	
Mod IF Glu + Insulin	457	0.1667	93	558	20.35%	↓^*^(Mod/Con)
HPB IF Glu + Insulin	758	0.1668	481	2,884	63.46%	↑^#^(Mod/HPB)
LPB IF Glu + Insulin	656	0.1668	389	2,332	59.30%	
MET-HCl IF Glu + Insulin	613	0.1668	205	1,229	33.44%	

*p < 0.05 vs. Con group; #*p* < 0.05 vs. Model group.

### 3.3 PB reshaped gut microbiota composition of the db/db mice

To investigate the effect of PB on gut microbiota of the db/db mice, 16S rRNA sequencing of the fecal samples was performed and the composition and abundances of gut microbiota were analyzed.

**Figure 3:**
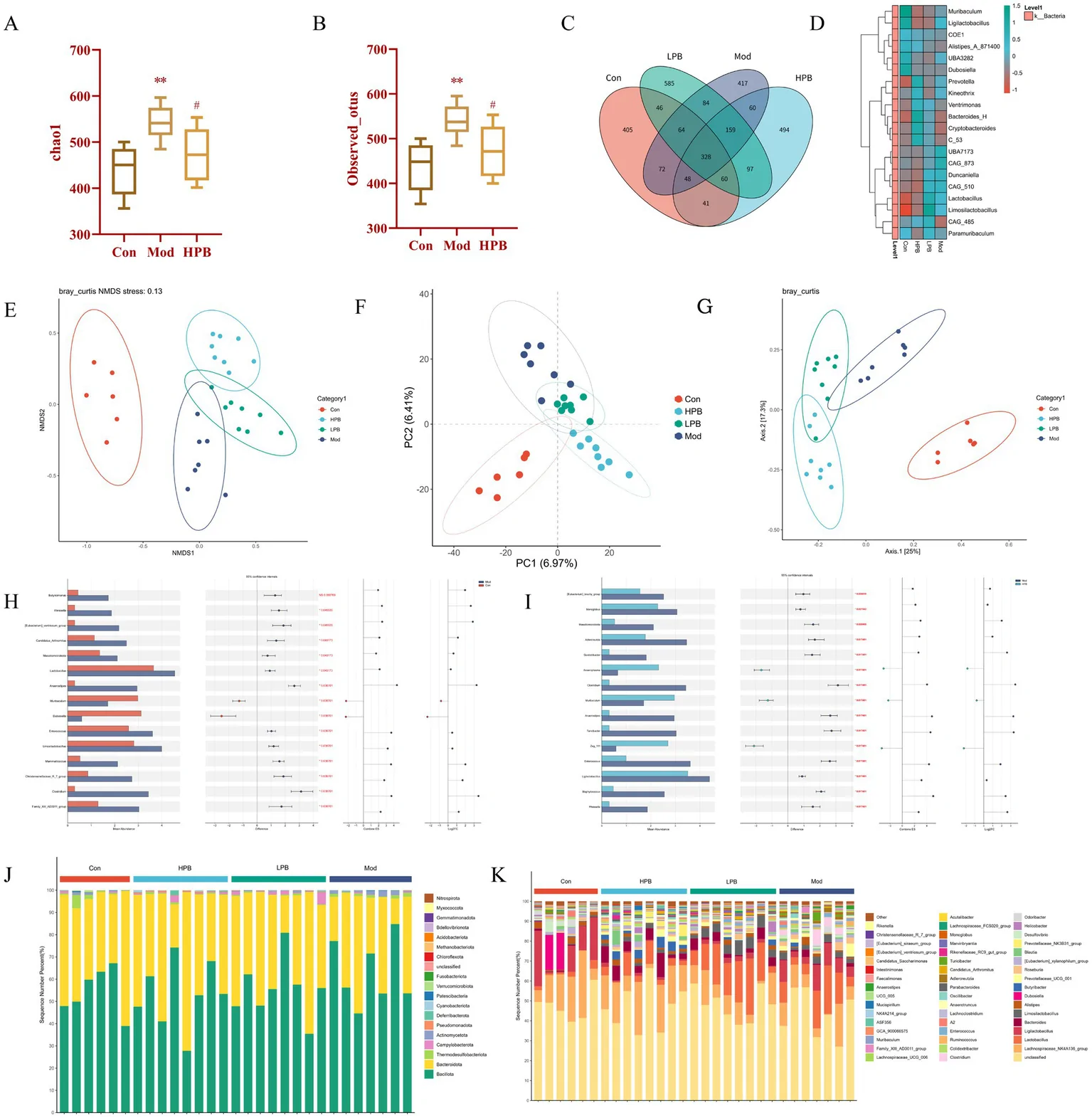
The results of microbial diversity analysis in PB efficacy experiment. **(A)** Chao1 indexes. **(B)** Observed OTUs indexes. **(C)** Venn diagrams of bacterial communities. **(D)** Heat map of gut microbiota. **(E)** PCoA analysis. **(F)** NMDS analysis. **(G)** PCA analysis. **(H)** Comparison of the function prediction results between the Con and Mod groups. **(I)** Comparison of the function prediction results between the Model and HPB groups. **(J)** Distribution histogram at the phylum level. **(K)** The distribution histogram of the genus level. **p < 0.0.01 vs. Con group; #*p* < 0.05 vs. Mod group. PERMANOVA analysis with 999 permutations was used to test intergroup microbial dissimilarity; all samples were rarefied to 42,000 valid reads prior to analysis, and rarefaction curves verified sufficient sequencing depth. Multiple testing was corrected by Benjamini–Hochberg FDR method.

The Chao1 and OTU indices (Figures 3A−B) which reflect microbial richness, were significantly elevated in the Mod group compared with the Con group (*p* < 0.05). However, the HPB group exhibited values comparable to those of the Con group. This suggested that PB intervention partially restored microbial richness in the T2DM model mice (Figure 3D).

Venn diagram analysis (Figure 3C) showed that 401 OTUs were shared between the Con, Mod, HPB, and LPB groups, but group-specific OTUs varied between the groups.

Principal coordinate analysis (PCoA) and Non-metric multidimensional scaling (NMDS) based on weighted UniFrac distance (Figures 3E−G) demonstrated clear separation of the microbial community structure between the 4 groups. The Mod and Con groups formed distinct clusters from the Con group, whereas the HPB and LPB groups showed a trend of shifting toward the Con group. This suggested that PB treatment partially restored the dysregulated gut microbiota structure in the T2DM mice.

Taxonomic profiling at the phylum and genus levels (Figures 3J,K) demonstrated significant alterations in microbial community structure in the Mod group relative to the Con group. Among all identified phyla, Firmicutes and Bacteroidetes exhibited the most pronounced alterations between groups. The Firmicutes/Bacteroidetes (F/B) ratio was significantly higher in the Mod group compared with the Con group, but this increase was significantly reversed by HPB treatment (*p* < 0.05). At the genus level (Figures 3H−I), Ligilactobacillus, Limosilactobacillus and Prevotellaceae-UCG showed the most significant changes between the groups; their relative abundances were significantly elevated in the Mod group, but HPB treatment significantly reduced them to levels comparable with the Con group.

### 3.4 PB reduces blood glucose in FMT-induced T2DM mice and reshapes the composition of their gut microbiota

**Figure 4:**
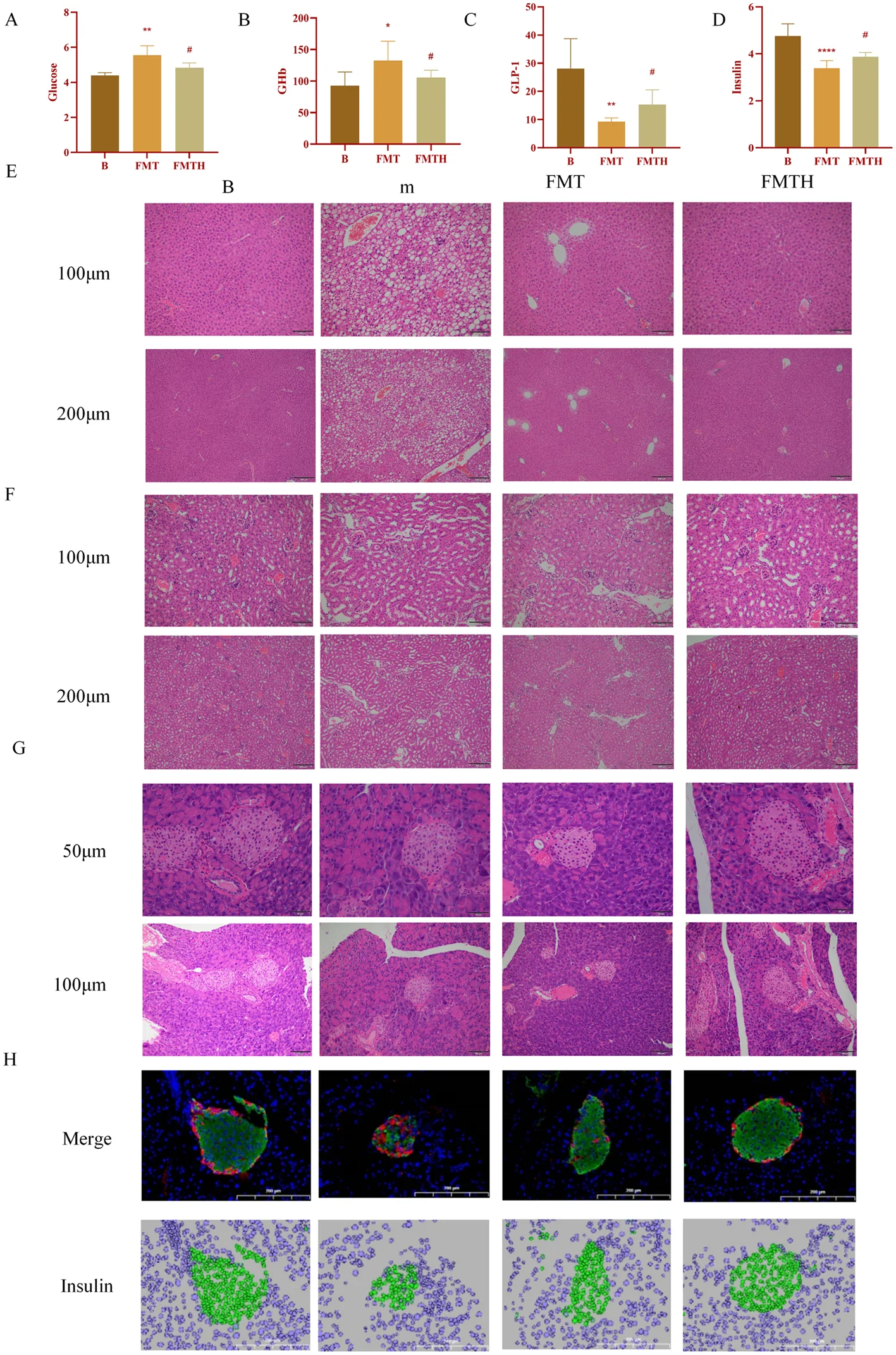
PB can reduce the outcome of T2DM induced by FMT. **(A)** Glucose. **(B)** GHb. **(C)** GLP-1. **(D)** Insulin. **(E)** Results of Oil Red O staining in liver. **(F–H)** HE staining results of liver, kidney, and pancreas. **(H)** Immunofluorescence results of pancreas. Immunofluorescence double staining of pancreas. Red: Insulin; Green: Glucagon. *p < 0.05, **p < 0.01, ***p < 0.001; #*p* < 0.05, ##*p* < 0.01.

Next, we investigated whether PB improves T2DM by regulating the gut microbiota environment. Toward this, we administered feces from “m” group mice into FMT group mice (normal C57BL/6 mice) to imbalance the gut microbiota in the FMT group and evaluated whether this imbalance led to hyperglycemia. Ten weeks later, mice in the FMTH group were administered PB. Four weeks after PB administration began, the fasting blood glucose of mice in the FMTH group was significantly lower than in the “m” group mice (*p* < 0.05). Compared with group B, the levels of GHb was significantly increased and the level of GLP-1 and insulin were significantly decreased in the FMT group mice (*p* < 0.05). Compared with the FMT group, the levels of GHb in the FMTH group mice was significantly decreased, and the level of GLP-1 and insulin were significantly increased (*p* < 0.05, Figures 4A–D). This confirmed the above findings.

Compared with group B, H&E and oil red O staining of liver tissue in group “m” showed obvious inflammatory infiltration, liver fibrosis and vacuolation. Furthermore, the degree of inflammatory infiltration in the liver tissue of mice in the FMT group was more obvious. H&E staining showed mild inflammatory cell infiltration in the kidneys, glomerular mesangial hyperplasia, and reduced number of pancreatic islets in the FMT group mice. Immunofluorescence staining of the pancreatic sections demonstrated a slight decrease in the expression of the target protein in the FMT group. The above symptoms were effectively abrogated in the FMTH group mice. This indicated that FMT imbalances composition of the gut microbiota and causes hyperglycemia, which affects several organ tissues. However, PB treatment in the FMTH group mice significantly alleviates FMT-induced liver and kidney damage (Figures 4E–G).

Glucagon and insulin double immunofluorescence staining was used to evaluate pancreatic *β*-cell positive ratio. The β-cell ratio in group B was normal at 35.58%, but was decreased significantly to 20.35% in group m, thereby indicating severe islet β-cell damage. The β-cell ratio increased slightly in the FMT group but remained lower than the baseline level. However, the β-cell ratio was restored to the normal baseline level in the FMTH group (35.60%). These results indicate that high-dose PB promotes pancreatic β-cell proliferation, repairs islet injury, and maintains functional homeostasis in the islets (Table 2; Figure 4H).

**Table 2:** Quantitative analysis of FMT pancreatic immunofluorescence.

Name/PN	DAPI TCS	Tissue area	Number of positive cells	Positive cell density	Positive cell ratio	Change trend
B IF Glu + Insulin	773	0.1678	275	1,639	35.58%	
m IF Glu + Insulin	457	0.1667	93	558	20.35%	↓^*^(m/B)
FMT IF Glu + Insulin	549	0.1667	170	1,020	30.97%	↓*(FMT/B)
FMTH IF Glu + Insulin	646	0.1667	230	1,380	35.60%	↑^#^(FMTH/FMT)

*p < 0.05 vs. Con group; #*p* < 0.05 vs. Model group.

### 3.5 PB reshaps the composition of the intestinal microbiota in FMT-induced T2DM mice

**Figure 5:**
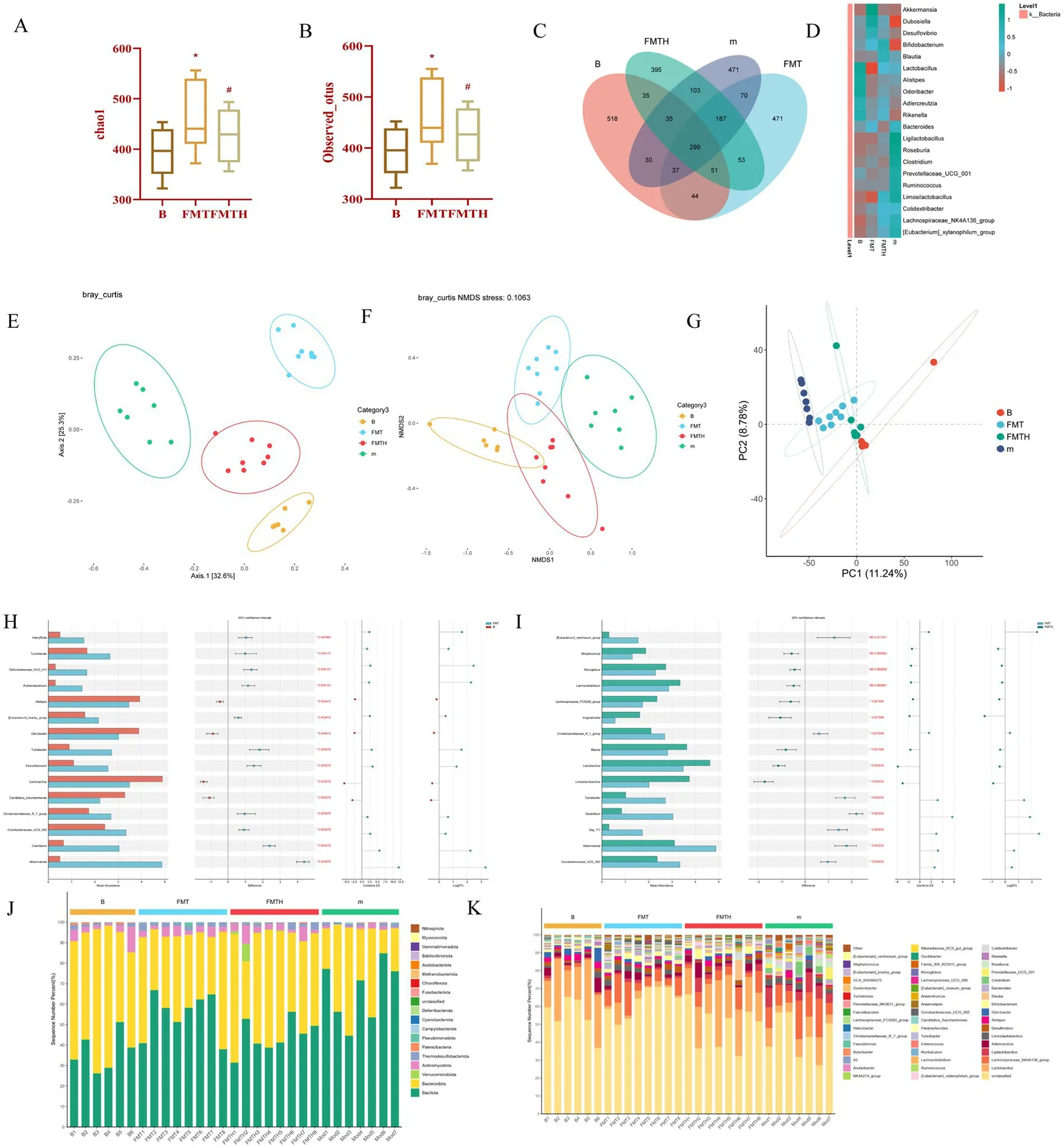
Effects of FMT with or without PB treatment on fecal microbiota in T2DM mice. **(A)** Chao1 indexes. **(B)** Observed OTUs indexes. **(C)** Venn diagrams of bacterial communities. **(D)** Heat map of gut microbiota. **(E)** PCoA analysis. **(F)** NMDS analysis. **(G)** PCA analysis. **(H)** Comparison of the function prediction results between the Con and Mod groups. **(I)** Comparison of the function prediction results between the Model and HPB groups. **(J)** Distribution histogram at the phylum level. **(K)** The distribution histogram of the genus level. *p < 0.05 vs. B group; #*p* < 0.05 vs. FMT group. PERMANOVA analysis with 999 permutations was used to test intergroup microbial dissimilarity; all samples were rarefied to 42,000 valid reads prior to analysis, and rarefaction curves verified sufficient sequencing depth. Multiple testing was corrected by Benjamini–Hochberg FDR method.

As shown in Figures 5A,B, the Chao1 and OTU indices were significantly elevated in the model group (m) and FMT group compared with the blank control group (B) (*p* < 0.05). This confirmed that FMT into C57BL/6 J mice successfully recapitulated the elevated microbial richness of the donor model state. In contrast, FMT combined with PB treatment (FMTH group) significantly reduced the Chao1 and OTU indices relative to the “m” and FMT groups and restored them to levels comparable to the B group (*p* < 0.05). These results indicate that the PB intervention (FMTH) effectively reverses increased microbial richness observed in T2DM model and FMT mice. Venn diagram analysis (Figure 5C) identified 209 shared OTUs between all four groups, as well as several distinct group-specific OTUs (unique OTUs: B group, 516; “m” group, 64; and FMT group, 70). This demonstrated successful transfer of model-like microbial community structure via FMT (Figure 5D). NMDS (Figures 5E–F) and PCoA (Figure 5G) based on Bray–Curtis distance demonstrated clear separation of microbial communities between the B group and the m/FMT groups. This confirmed that the FMT mice clustered closely with the model mice and were distinct from the control mice. The FMTH group, however, clustered more closely with the B group. This suggested that FMT combined with PB treatment effectively restores the dysregulated gut microbiota structure in the T2DM mice. Taxonomic profiling at the phylum and genus levels (Figures 5J,K) demonstrated significant shifts in the microbial community structure in the “m” and FMT groups compared with the B group. The data showed consistent patterns of dysregulation between these two groups. Linear discriminant analysis Effect Size (LEfSe) analysis (Figures 5H,I) identified key differentially abundant taxa among the groups. Compared with the B group, FMT group exhibited significantly increased relative abundances of *Lactobacillus*, *Enterococcus*, *Muribaculum*, and *Akkermansia*. This demonstrated successful establishment of model-like microbiota via FMT. FMTH treatment significantly reduced the abundances of *Lactobacillus*, *Enterococcus*, and *Akkermansia* compared with the “m” and FMT groups, whereas FMT alone maintained the dysregulated microbial profile of the model state. These findings demonstrate that FMT combined with PB treatment normalizes the dysregulated microbial composition in the T2DM mice, especially by suppressing the overgrowth of pathogenic or inflammation-associated genera.

### 3.6 16S rRNA gene sequencing of *in vitro* fermentation combined with PB treatment

**Figure 6:**
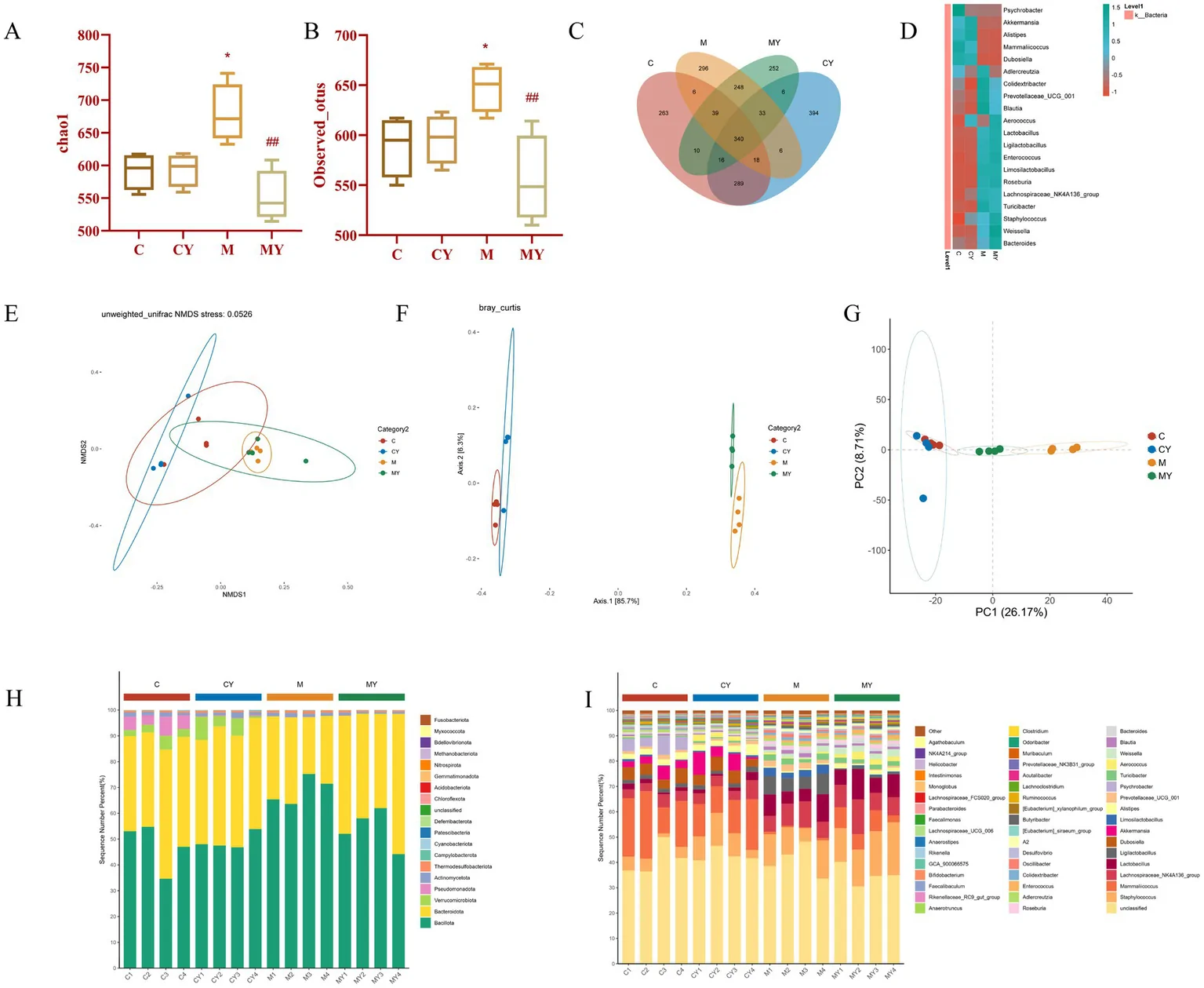
The results of microbial diversity of *in vitro* fermentation. **(A)** Chao1 indexes. **(B)** Observed OTUs indexes. **(C)** Venn diagrams of bacterial communities. **(D)** Heat map of gut microbiota. **(E)** PCoA analysis. **(F)** NMDS analysis. **(G)** PCA analysis. **(H)** Distribution histogram at the phylum level. **(I)** The distribution histogram of the genus level. *p < 0.05 vs. C group; ##*p* < 0.01 vs. M group. PERMANOVA analysis with 999 permutations was used to test intergroup microbial dissimilarity; All samples were rarefied to 42,000 valid reads prior to analysis, and rarefaction curves verified sufficient sequencing depth. Multiple testing was corrected by Benjamini–Hochberg FDR method.

As shown in Figures 6A,B, the Chao1 and OTU indices were elevated in M compared with C, but were partially reversed in MY, thereby restoring the indices to levels closer to C and CY (*p* < 0.05). No significant differences in *α*-diversity were observed between C and CY. This suggested that PB did not significantly impact the microbial richness of healthy fecal microbiota under *in vitro* fermentation conditions. Venn diagram analysis (Figure 6C) identified 268 shared OTUs among all four groups, as well as distinct group-specific OTUs (unique OTUs: C, 245; CY, 308; M, 15; and MY, 5). This highlighted significant differences in the microbial community structure between control and model states under *in vitro* fermentation. LEfSe analysis (Figure 6D) identified key differentially abundant taxa between the 4 groups. NMDS (Figure 6E) and PCoA (Figure 6G) based on Bray–Curtis distance demonstrated clear separation of microbial communities between C, CY and M, MY. Furthermore, MY showed a trend of shifting toward the control groups. This suggested that co-culturing with PB partially restores the dysregulated gut microbiota structure in M under *in vitro* fermentation conditions. Taxonomic profiling at the phylum and genus levels (Figures 6H,I) revealed significant shifts in the microbial community structure in the M group compared with the C group.

### 3.7 Mechanism by which PB mitigates T2DM through fecal metabolomics

**Figure 7:**
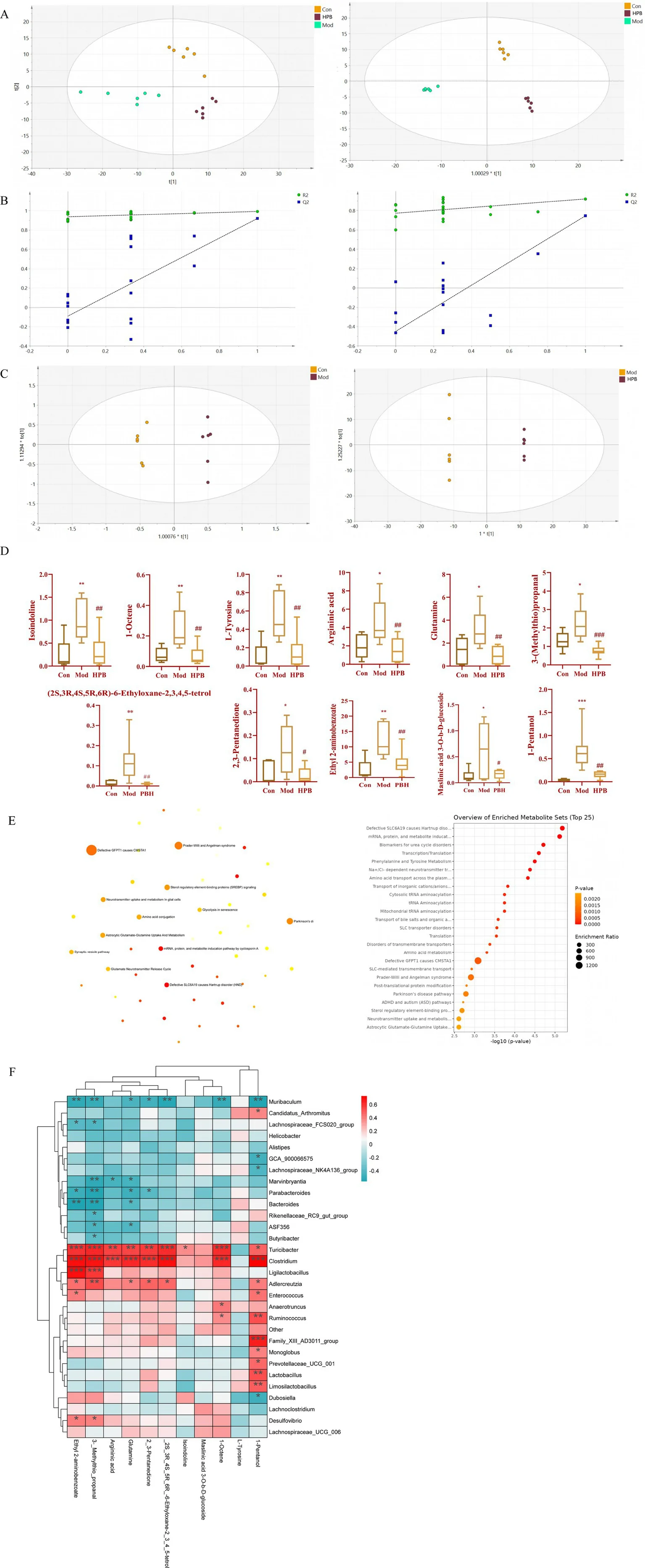
Multivariate statistical analysis of serum metabolomics. **(A)** PLS-DA score plot. **(B)** PLS-DA replacement analysis. **(C)** OPLS-DA plots. **(D)** Identified 16 potential biomarkers regulated by PB. **(E)** Pathway enrichment analysis. **(F)** Correlation analysis. Differential metabolites were screened by *VIP* > 1.0 and FDR-adjusted *p* < 0.05; 200 permutation tests were conducted to verify OPLS-DA model robustness. All metabolite signals were normalized by total ion current, and instrument stability was validated via repeated QC samples with peak area *RSD* < 15%. *p < 0.05, **p < 0.01, ***p < 0.001.

Furthermore, orthogonal partial least squares discriminant analysis (OPLS-DA) was performed to delineate the global metabolic signatures across the Con, Mod and HPB groups (Figures 7A,C). Permutation testing verified the robustness of the constructed OPLS-DA models, with no evident overfitting observed (Figure 7B). Variable importance in projection (VIP) screening was subsequently conducted with predefined thresholds of VIP > 1.0 and *p* < 0.05, which identified a total of 11 markedly perturbed metabolites, including isoindoline, 1-octene, L-tyrosine, argininic acid, glutamine, 3-(methylthio)propanal, (2S,3R,4S,5R,6R)-6-ethyloxane-2,3,4,5-tetrol, 2,3-pentanedione, ethyl 2-aminobenzoate, maslinic acid 3-O-*β*-D-glucoside and 1-pentanol. Box plot visualization illustrated that the abundances of these metabolites were profoundly dysregulated in the Mod group, whereas HPB intervention significantly restored their aberrant levels to a physiological state comparable to the Con group (Figure 7D).

Metabolic pathway enrichment analysis implemented via MetaboAnalyst 6.0 revealed that these differential metabolites were predominantly enriched in pathways governing inorganic cation/anion transmembrane transport, amino acid metabolism, glutamate-glutamine turnover, solute carrier (SLC) transporter disorders, as well as Parkinson’s disease-associated metabolic cascades (Figure 7E). Collectively, these results indicated that HPB alleviates T2DM-related metabolic dysfunction by modulating ion transmembrane transport, amino acid homeostasis and multiple small-molecule secondary metabolic pathways.

Genus-metabolite correlation heatmaps further uncovered extensive correlative interactions between gut bacterial genera and characteristic differential metabolites (Figure 7F). Diabetes-enriched genera including Clostridium, Ligilactobacillus and Adlercreutzia exhibited strong positive correlations with amino acid metabolites (argininic acid, glutamine and L-tyrosine, *p* < 0.001), highlighting their vital functional roles in intestinal nitrogen metabolism. In contrast, Bacteroides, Parabacteroides, Muribaculum and Marvinbryantia displayed prominent negative correlations with alkane and lipid metabolites (1-octene, 1-pentanol and isoindoline), implying that lipid accumulation under diabetic conditions may suppress the proliferation of these commensal bacteria. Additionally, Prevotella, Lactobacillus and Dubosiella were positively correlated with maslinic acid 3-O-*β*-D-glucoside and ethyl 2-aminobenzoate, two metabolites drastically upregulated upon HPB treatment. Hierarchical clustering segregated gut microbes and metabolites into discrete clusters, which reflected the intrinsic functional coordination within the gut microbiota-metabolite interactome.

## 4 Discussion

T2DM is a global public-health challenge with increasing prevalence, especially among the younger populations. Yet, conventional medicines are often limited by single-target mechanisms and significant adverse effects (Reaven and Salans, 1964; Brody, 2012). In traditional Chinese medicine (TCM), T2DM is classified as “Xiaoke syndrome,” which is characterized by yin deficiency and internal heat, thereby supporting the use of heat-clearing herbs such as PB (Chen et al., 2023).

Several studies have established that flavonoids are the principal bioactive constituents of many medicinal plants. They exert antidiabetic effects, including glycemic control and improvement of insulin sensitivity, through multiple pathways (Guo et al., 2019). Moreover, gut microbiota has emerged as a critical regulator of host glucose metabolism. T2DM patients exhibit distinct microbial dysbiosis that can be modulated by herbal interventions to enhance secretion of incretin hormones such as GLP-1 (Aron-Wisnewsky et al., 2021; Tomasics et al., 2023; Wu et al., 2024).

Compared with previous studies on PB against T2DM, the present study made significant advances regarding the antidiabetic role of PB. First, we focused on total flavonoids and conducted a comprehensive UHPLC-HRMS characterization. This resulted in the identification of 41 compounds, including 8 flavonoids, 9 flavonols and 2 dihydroflavonoids, thereby clarifying the chemical basis of the antidiabetic action of PB. Second, we performed descriptive microbiome analysis, FMT, and *in vitro* co-fermentation experiments and provide causal evidence that PB improves T2DM by modulating the gut microbiota. Third, we performed integrated fecal non-targeted metabolomics and identified 15 differential metabolites and key pathways, including glucose homeostasis as well as bile acid and amino acid metabolism. This links alterations in the gut microbiota to restoration of functional metabolism. Collectively, these multi-omics and causal validation approaches establish a more comprehensive mechanism for PB. Our data shows that total flavonoids of PB is a promising prebiotic-like candidate for T2DM management.

The identification of 41 compounds in PB, including 8 flavonoids, 9 flavonols, and 2 dihydroflavonoids, highlights the chemical complexity underlying its pharmacological effects. Consistent with previous reports, flavonoid-rich extracts from medicinal plants often exhibit multitarget activities against metabolic disorders (Dai et al., 2018). For example, we identified apigenin and luteolin, which have previously been associated with improved glucose homeostasis and reduced insulin resistance by modulation of inflammatory pathways and AMPK activation. Furthermore, flavonols such as kaempferol and quercetin have been shown to enhance GLP-1 secretion and preserve pancreatic *β*-cell function (Olasehinde and Olaokun, 2024; Wang et al., 2025).

Pharmacodynamic data demonstrated that PB treatment significantly reduced blood glucose and GHb levels, increased insulin and GLP-1 secretion, and ameliorated liver, kidney, and pancreatic damage in db/db mice. These findings align with previous findings that natural flavonoids improve pancreatic function and mitigate diabetes-associated organ injury (Li Y. et al., 2024). Furthermore, elevation of GLP-1 following PB administration suggests a potential mechanism involving the enteroendocrine axis, a pathway increasingly recognized as a key target for antidiabetic therapies (Madsbad and Holst, 2025).

Gut microbiota are pivotal mediators of the metabolic effects of dietary and herbal components (Che et al., 2022). In the present study, 16S rRNA sequencing showed that the intestinal microbiota of db/db mice was dominated by *Firmicutes* and *Bacteroidetes* at the phylum level. Lactobacillus species exert complex regulatory effects on glycolipid metabolism and inflammatory responses. A recent review (Ren et al., 2025) systematically validates lactic acid bacteria as effective preventive and therapeutic candidates for T2DM. Their core protective function involves mitigating excessive oxidative stress, thereby stabilizing glycolipid metabolism and suppressing inflammatory hyperactivation.

Notably, PB treatment reversed these dysbiotic changes, restoring the relative abundances of these key genera toward those observed in healthy control mice. Similar modulatory effects have been reported for other flavonoid-rich herbal extracts, such as those from *Coptis chinensis* and *Rehmannia glutinosa*, which improve metabolic outcomes by reshaping gut microbiota composition (Wang et al., 2023; Lv et al., 2023).

The functional relevance of these microbial changes was further substantiated by FMT experiments, wherein transplantation of microbiota from T2DM mice induced hyperglycemia and metabolic dysfunction in the recipient mice, but PB administration effectively ameliorated these effects. This causal evidence supports the notion that PB exerts its antidiabetic effects at least in part by modulating the gut microbiota. FMT has been used to establish microbiota-dependent mechanisms previously (Zhang et al., 2020).

Furthermore, *in vitro* co-fermentation experiments demonstrated direct interactions between PB and gut microbiota, leading to the generation of bioactive metabolites, which may contribute to the overall therapeutic efficacy. This biotransformation by intestinal bacteria is a critical step for many natural glycosides, which undergo deglycosylation and other metabolic conversions to produce more readily absorbable and biologically active aglycones.

Fecal metabolomics identified 15 differential metabolites between normal and T2DM mice. This provides insight into the metabolic pathways perturbed by T2DM and restored by PB treatment. In T2DM, the levels of multiple metabolites in the model-group mice are significantly elevated. The reasons for this can be attributed to three plausible reasons: First, insulin resistance and glucolipid metabolic disorders lead to abnormalities in glycolysis, fatty acid oxidation, and amino acid catabolic pathways, resulting in the accumulation of glucose metabolic intermediates, incompletely oxidized fatty acid derivatives, and branched-chain and aromatic amino acids in the body (Accili et al., 2025; Yazıcı and Sezer, 2017). Second, gut microbiota dysbiosis leads to an increased abundance of pathogenic bacteria. These bacteria produce or promote host absorption of microbiota-related metabolites, including bile acid derivatives and trimethylamine, leading to increased levels of these compounds in the blood or tissues (Xu et al., 2025; Wei et al., 2024). Third, under disease conditions, liver detoxification function and kidney excretion capacity are impaired due to steatosis, diabetic nephropathy, and other damages, leading to reduced clearance of metabolites, thereby exacerbating the accumulation effect (Zhang et al., 2010; Zhao et al., 2009).

In contrast, normal control mice maintain metabolite levels at a relatively low and balanced state because of their intact metabolic regulatory system and gut microbiota homeostasis, as well as normal liver and kidney function that effectively control the production and clearance of metabolites. The PB treatment group shows improved insulin resistance, reshaped gut microbiota structure, and mitigated liver and kidney damage. This restores the body’s ability to utilize and excrete metabolites, thereby significantly reducing the levels of these abnormally elevated metabolites close to those in the normal control group.

These metabolites were predominantly involved in pathways related to glucose homeostasis, bile salt metabolism, amino acid metabolism, and transport of inorganic cations and anions. Alterations in bile acid metabolism are closely linked to T2DM pathogenesis with bile acids acting as signaling molecules regulating glucose and energy metabolism through the farnesoid X receptor and TGR5 pathways (Prawitt and Staels, 2010). Similarly, amino acid metabolites such as L-tyrosine and glutamine are implicated in insulin resistance and *β*-cell dysfunction, and their dysregulation is often observed in diabetes (Zhu et al., 2022).

Reversal of these metabolite aberrations by PB suggests that its beneficial effects extend beyond glycemic control and involves broader metabolic restoration. The integrated approach combining microbiome and metabolomic analyses offers a powerful means to dissect the complex interplay between host metabolism and gut microbiota in response to herbal interventions.

**Figure 8:**
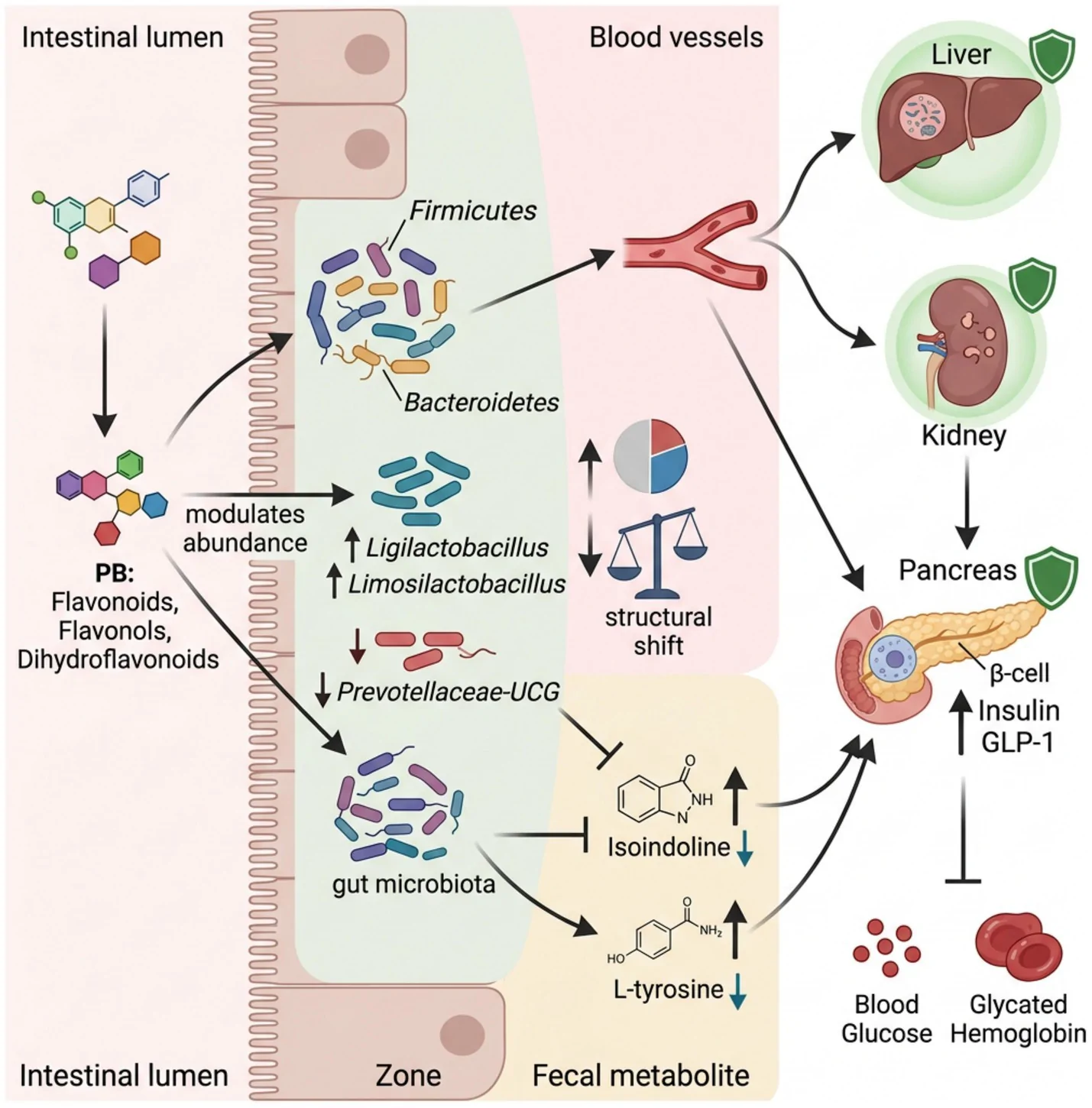
Mechanism diagram of PB treatment for T2DM.

Collectively, these findings demonstrate that PB improves T2DM by modulating gut microbiota composition, enhancing GLP-1 secretion, and restoring metabolic homeostasis, thereby providing a mechanistic basis for its therapeutic application (Figure 8).

## 5 Conclusion

This study systematically investigated the ameliorative effects of PB on T2DM and its underlying mechanisms. The results demonstrated that PB contains multiple flavonoid components, which significantly reduce fasting blood glucose and GHb levels, increase insulin and GLP-1 secretion, and effectively alleviate liver, kidney, and pancreatic damage in the db/db mice. Furthermore,16S rRNA sequencing, FMT, and *in vitro* cofermentation experiments confirmed that total flavonoids of PB modulate the gut microbiota structure and reverse the aberrant changes in model mice. Fecal metabolomics further revealed that intervention with total flavonoids of PB regulated multiple differential metabolites involved in pathways related to glucose metabolism, bile acid metabolism, and amino acid metabolism. Collectively, PB exerts anti-T2DM effects by modulating gut microbiota homeostasis, promoting GLP-1 secretion, and restoring metabolic balance, thereby providing a scientific basis for its clinical application.

## Data Availability

The datasets presented in this study can be found in online repositories. The names of the repository/repositories and accession number(s) can be found in the article/Supplementary material.
